# Association between dietary inflammatory potential and the probable sarcopenia among community-dwelling older adults: a cross-sectional study

**DOI:** 10.1186/s12877-022-03525-2

**Published:** 2022-11-03

**Authors:** Zahra Esmaeily, Shahrzad Daei, Mahshid Rezaei, Atefeh Eyvazkhani, Zahra Tajary, Marjan Mansouri Dara, Ahmad Reza Dorosty Motlagh

**Affiliations:** 1 Department of Community Nutrition, School of Nutritional Sciences and Dietetics, Tehran University of Medical Sciences, Tehran, Iran; 2 Department of Nutrition, Science and Research Branch, Islamic Azad University, Tehran, Iran

**Keywords:** Aging, Dietary inflammatory index, Empirical dietary inflammatory index, Handgrip strength, Probable sarcopenia

## Abstract

**Background:**

Unlike the numerous studies concerning the role of dietary inflammatory potential in chronic diseases, limited studies focused on the association of dietary inflammatory potential with handgrip strength (HGS) and probable sarcopenia (PS). This study tends to explore the association between dietary inflammatory potential and PS among older adults in Tehran.

**Methods:**

The cross-sectional study was conducted between May and October 2019 on 201 randomly selected older adults in Tehran, Iran. A validated food frequency questionnaire was utilized for recording dietary intake. Dietary habits were evaluated through Dietary Inflammatory Index (DII) and Empirical Dietary Inflammatory Index (EDII). PS assessment was done by HGS estimation. Statistical evaluation included descriptive analyses, logistic, and linear regression.

**Results:**

Those probably suffering from sarcopenia were older than healthy ones (*p* < 0.0001) and had significantly higher DII scores (*p* = 0.05) but not EDII (*p* = 0.85). Besides, PS subjects had a lower intake of anti-inflammatory nutrients. The odds of PS were doubled in people on the top tertile of DII (OR = 2.49, 95% (CI) = 1.11–5.58) and second tertile of EDII (OR = 2.29, 95% (CI) = 1.03–5.07) relative to bottom tertiles after adjusting for confounders. The relationships between index scores and HGS were simply significant in the adjusted model of EDII and HGS (B = -0.49, *p* = 0.04).

**Conclusion:**

Conclusively, participants adhering to a pro-inflammatory diet had more likelihood of PS. Findings are in line with current recommendations to reduce unhealthy foods with more inflammatory potential. These findings warrant confirmation in high-quality interventional studies.

## Introduction

Physical changes happen gradually as a consequence of aging. Loss of muscle strength and muscle mass are the most prevalent modifications after age 50 [1]. The reduction of 3% muscle strength and 1% muscle mass happens annually in adulthood which is the pathologic form of the decline called sarcopenia [2, 3]. Sarcopenia is a multifactorial, age-dependent disorder associated with a sedentary lifestyle and malnutrition [1]. Sarcopenia has different causes, which include age-related factors like decreased physical activity, anorexia of aging, low vitamin D, weight loss, and elevated pro-inflammatory cytokines [4]. In 2018, the European Working Group on Sarcopenia in Older People (EWGSOP) defined probable sarcopenia (PS) by low muscle strength as a powerful predictor of sarcopenia [5]. Muscle weakness increases the odds of falling and causes serious injuries in different parts of the body [6]. It could also predict poor patient outcomes e.g. prolonged hospitalization, poor health-related quality of life, and death, and is a frailty marker that increases the possibility of mobility limitation [5, 7, 8]. Sarcopenia progression could be prevented by the assessment of PS to provide applicable information about sarcopenia. In the current situation of global aging, the future increase in sarcopenia health costs is evident and some interventions have been required to decrease the loss of muscle mass or restore it in older adults [9]. Although the loss of muscle mass and decrease of muscle strength could occur due to aging, different grades of this reduction have been observed in the population. It shows that changeable habits like diet may have a role in the progression of sarcopenia [10, 11].

Assessing diet quality is one of the ways to demonstrate a person’s diet status. The Dietary Inflammatory Index (DII) and very recently Empirical Dietary Inflammatory Index (EDII) was developed by Shivappa (a priori) and Tabung et al. (a posteriori) to assess the inflammatory potential of a dietary pattern. A high score of these indexes has a significant association with increased serum and blood inflammatory markers [12, 13]. Furthermore, they evaluate the association between diet quality and chronic inflammatory outcomes like metabolic and pulmonary diseases, cancer, and fractures [14–17].

Several studies have considered the association of DII and EDII with a risk of different morbidities. Post-menopausal women with a high risk of osteoporosis tend to have a higher score of DII which indicates a pro-inflammatory diet [18]. The further risk of cardiovascular disorder, metabolic syndrome, hyperglycemia, and abdominal obesity were associated with a pro-inflammatory diet [16, 19]. The high risk of frailty was associated with a high score of DII in older adults [20]. On the other hand, few studies have focused on the association between dietary inflammatory potential and muscle weakness or sarcopenia. The pro-inflammatory diet, evaluated by DII, leads to high odds of sarcopenia and osteosarcopenic obesity [21, 22]. Low gait speed and increased risk of fractures were associated with the inflammatory potential of diet [17]. Cervo et al. suggested that a pro-inflammatory diet might be harmful to musculoskeletal health in men relative to women [23].

A rapid growth in the Iranian elderly population from 6.4% to 20.2% within 2019–2050 [24] turns the age-related complications (i.e. PS and sarcopenia) into a nationally important issue that needs particular emphasis. Despite the investigation of the association between dietary inflammatory potential and sarcopenia in various studies, none has assessed the association with PS. Hence, this cross-sectional study aimed to consider the association of dietary inflammatory potential, evaluated by DII and EDII, with PS in older residents of Tehran, Iran.

## Methods and materials

This cross-sectional study was carried out on 201 randomly-selected older residents (60 years old ≤) of Tehran, Iran between May and October 2019. The sample size was defined according to type I error of α = 0.05 and type II error of β = 80%, thus, 191 overall subjects were needed for this study. Finally, 201 participants were included to further increase statistical power. Those with energy intake between 800–4200, no changes in their dietary habits over the last year, walking without any helping equipment, prosthetic or artificial limbs, and without an acute form of any disease were entered in the present study. For sampling, Tehran was divided into 5 regions: east, west, north, south, and city center. Details of the sampling process were described elsewhere [25]. After taking written consent from participants, their demographic and socioeconomic information, physical activity, and medical history were questioned by a standardized questionnaire. The physical activity was the amount of daily average time used to exercise, jog or do other sports which were estimated by participants. Socioeconomic status was defined by collecting data about education and economic state. Considering the possibility of refusals for declaring monthly income, a 9-item questionnaire (possession of house, car, side-by-side refrigerator, washing machine, dishwasher, laptop/personal computer, sofa, microwave, and handmade carpet) [26] was used in addition to querying about the house and car ownership for quantifying the economic status. The subjects’ economic status was classified as: Very bad: ≤ 3 items without any personal home and car. Bad: ≤ 3 items with personal home or car. 4–6 items without personal home and car. Average: 4–6 items with personal home or car. 7 items ≤ without a personal home and car. Good: 7 ≤ items with personal home or car. Very good: 7 items ≤ with personal home and car.

This study was approved by the ethics committee of Tehran University of Medical Sciences. The protocol number of the local ethics committee was IR.TUMS.VCR.REC.1398.476.

### Anthropometric measurements

Waist, hip, and arm circumferences, weight, and height were measured in the current study. Body mass index (BMI), waist-to-hip ratio (WHR), and waist-to-height ratio (WHtR) were calculated in regard to the aforementioned measures. Weight (kg) and height (cm) were measured with light clothes and without shoes on with a Camry EB9011 scale (Camry Co, Zhongshan, China) and a Fiber-Glass tape measure, respectively. The measurement of a midpoint between the lower edge of the chest and the upper edge of the iliac crest and the last rib formed waist circumference (WC), and hip circumference was the maximum circumference of the hip when a participant stood firmly. The mid-upper arm circumference (MUAC) was the measured circumference of the scapular and olecranon midway of the non-dominant hand with the elbow flexed 90°. Circumferences were measured by a Fiber-Glass tape measure as well.

Participants with BMI lower than 23.5 and higher than 30.9 kg/m^2^ were underweight and obese, respectively [27]. Abdominal obesity was specified as waist circumference higher than 88 cm and 102 cm in women and men, respectively. Moreover, people with WHtR ≥ 0.6 and women with a WHR higher than 0.85 were classified as abdominally obese as well [28, 29]. Notably, WHR was not used for men due to cultural and religious matters.

### Dietary data collection

A previously validated semi-quantitative food frequency questionnaire (FFQ) was utilized with 147 items for estimation of the usual dietary intake throughout last year [30]. Major items contained intake of bread and grains, legumes, meat, and meat-derived products, poultry, fish, eggs, dairies, kinds of butter, vegetables, pickles, fruits and fruit juices, oils, seeds, and nuts, added sugar, drinks, spices, and salt. The frequencies and portion sizes of each item were asked. Finally, the dietary intake quality was assessed by DII and EDII. Questionnaires were completed by trained dietitians.

### Calculation of Dietary Inflammatory Index

DII was determined according to the approach suggested by Shivappa et al [12]. Considering the usage of the 147-item FFQ, 29 out of 45 components of DII were scored in this study which includes 24 nutrients, onion, garlic, turmeric, pepper, and tea. The DII scoring procedure is as follows: 1) Each component’s Z-score has been calculated based on the global mean and standard deviation which has been reported elsewhere [12], 2) The Z-score was converted to the percentile to minimize the effect of the right skewing, 3) The percentile value doubled and subtracted by 1 for computing the centered percentile, 4) Multiplying the centered percentile by the overall inflammatory effect score made each parameter’s DII score. Finally, the sum of all derived values forms the overall DII score.

### Calculation of Empirical Dietary Inflammatory Index

Eighteen food groups form EDII following the Tabung et al. system [13] which wine and beer were not used to make EDII in the present study due to religious reasons. High- and low-energy beverages were considered as one food item in the FFQ, thus, 15 food parameters included in this study as inflammatory (processed meat, red meat, organ meat, other fish, other vegetables, refined grains, high-energy beverages, and tomatoes) and anti-inflammatory (dark yellow vegetables, leafy green vegetables, snacks, fruit juice, pizza, tea, and coffee) categories with more positive and negative scores, respectively. The mean daily intake of each food group was identified by defined serving sizes and weighted by the proposed regression coefficients. The weighted food group intakes were summed to constitute EDII and rescaled by dividing by 1000 to reduce the magnitude of the score for facilitating the interpretation.

### Probable sarcopenia

As stated by EWGSOP2, handgrip strength (HGS) was evaluated as a surrogate measurement of muscle strength to determine PS [5]. A squeeze dynamometer (Saehan SH5008, Co, Seoul, Korea) was used as the HGS determinant. Participants sat on a chair with the arm bent at 90°; were asked to squeeze the dynamometer 3 times with the extreme force of each hand and held it for 10 s with 30 s rest between every attempt. Eventually, the average maximum power of each hand was ascertained as the participant’s HGS. Since the aforementioned dynamometer has not been used in former studies, the accuracy of the dynamometer was checked against a Jamar dynamometer, the gold standard for testing HGS [3]. The results of the squeeze dynamometer would comparable with the Jamar dynamometer if the amounts are multiplied by 1.6. Thereby, participants were defined to have a high probability of sarcopenia when the HGS was < 10 kg (women) and < 16.8 kg (men) in the present study.

### Statistical analysis

DII and EDII were divided into tertiles to assess dietary quality. Normality distribution was checked using Kolmogorov–Smirnov’s test. Independent Student’s *t*-test and ${x}^{2}$ test was applied respectively to determine the significant differences of quantitative (Mean ± standard deviation (SD)) and qualitative variables (frequencies (%)) between the two groups (probably sarcopenic and non-sarcopenic). Age, gender, CVD medication, BMI, family number, and physical activity were adjusted to compare the mean-dependent variables by analysis of covariance (ANCOVA). A multiple linear regression model was performed to adjust for confounders of HGS to assess the actual relationship of DII and EDII with HGS. Finally, binary logistic regression was utilized for evaluating the association of adherence to DII and EDII with PS by adjusting the above-mentioned covariates. Statistical significance α was accepted at 0.05. Statistical Package for Social Sciences (SPSS Inc., Chicago, IL, USA, version16) was used for all statistical analyses.

## Results

### Participant Characteristics

A total of 46 men (23%) and 155 women (77%) with a mean age of 66 years (ranging from 60 to 85) were included in this study. They had a daily physical activity of 32 min and a BMI of 29 kg/m^2^. The most common diseases among participants were cardiovascular diseases and skeletal disorders. PS subjects were older than healthy ones (67 vs 64 years, *p* < 0.0001) and constitute 61% of the total study population. Additionally, they had low MUAC (*p* = 0.02) and a worse economic state in relation to subjects with normal HGS (*p* = 0.002). The summary of the main characteristics of these participants was demonstrated in Table 1.

**Table 1:** Participant characteristic

Variables	Non-sarcopenic (*N*=78)		Probable Sarcopenia (*N*=123)		*P*-value*
	Mean	SD	Mean	SD	
Age (year)	63.9	3.66	67.54	5.94	<0.0001
Postmenopausal age (year)	47.35	4.86	47.91	5.64	0.53
Physical activity (min)	37.20	42.3	29.64	42.3	0.07
Weight (kg)	74.16	9.96	72.2	11.56	0.22
Height (m)	1.59	0.08	1.58	0.09	0.17
Waist circumference (cm)	97.67	8.92	97.28	10.38	0.79
MUAC (cm)	32.5	2.09	31.56	3.01	0.02
Body mass index (kg/m^2^)	29.26	4.07	28.82	4.09	0.45
WHtR	0.61	0.07	0.61	0.08	0.7
WHR ^a^	0.88	0.06	0.86	0.1	0.25
HGS (Kg)	13.29	3.52	9.16	3.03	<0.0001
	N (%)		N (%)		
Gender					0.12
Male	14 (30.4)		32 (69.6)		
Female	64 (41.3)		91 (58.7)		
Marital status					0.16
Married	61 (41.8)		85 (58.2)		
Other	17 (27.33)		38 (72.67)		
Head of the family					0.18
Father	15 (32.6)		31 (67.4)		
Mother	62 (41.9)		86 (58.1)		
Education					0.14
High school or lower	52 (36.1)		92 (63.9)		
University	26 (45.6)		31 (54.4)		
Economic status					0.002
Very bad	7 (21.2)		26 (78.8)		
Bad	20 (48.8)		21 (51.2)		
Average	9 (25)		27 (75)		
Good	9 (26.5)		25 (73.5)		
Very good	32 (57.1)		24 (42.9)		
Supplements					
Vitamin D	56 (40.9)		81 (59.1)		0.24
Multivitamins	32 (41)		46 (59)		0.36
Minerals	39 (39)		61 (61)		0.52
Disorders					
Diabetes	19 (38)		31 (62)		0.52
Cardiovascular	33 (29.5)		79 (70.5)		0.004
Pulmonary	7 (35)		13 (65)		0.46
Renal	4 (21.1)		15 (78.9)		0.07
Skeletal	46 (34.8)		86 (65.2)		0.08
Psychological	19 (31.7)		41 (68.3)		0.12
Medication					
Diabetes	14 (33.3)		28 (66.7)		0.28
Cardiovascular	25 (26)		71 (74)		<0.0001
Skeletal	17 (32.7)		35 (67.3)		0.2
Psychological	11 (42.3)		15 (57.7)		0.41
BMI Status					0.49
Underweight	4 (36.4)		7 (63.6)		
Normal	50 (38.8)		79 (61.2)		
Overweight	24 (39.3)		37 (60.7)		
WC Status.					0.11
Normal	17 (30.9)		38 (69.1)		
Abdominal obesity	61 (41.8)		85 (58.2)		
WHtR status					0.19
Normal	31 (36.9)		53 (63.1)		
Abdominal obesity	47 (40.2)		70 (59.8)		
WHR status ^a^					0.38
Normal	23 (39)		36 (61)		
Abdominal obesity	41 (42.7)		55 (57.3)		

*HGS* Handgrip strength, *SD* standard deviation, *BMI* body mass index, *WC* waist circumference, *MUAC* Mid-upper arm circumference, *WHtR* Waist- to-height ratio, *WHR* Waist-to-hip ratio

*P ≤ 0.05; Student’s t-test was used for comparing the means difference of quantitative variables, X^2^ test was used for qualitative variables

^a^ Calculated in women

### Dietary inflammatory potential and hand grip strength

**Fig. 1:**
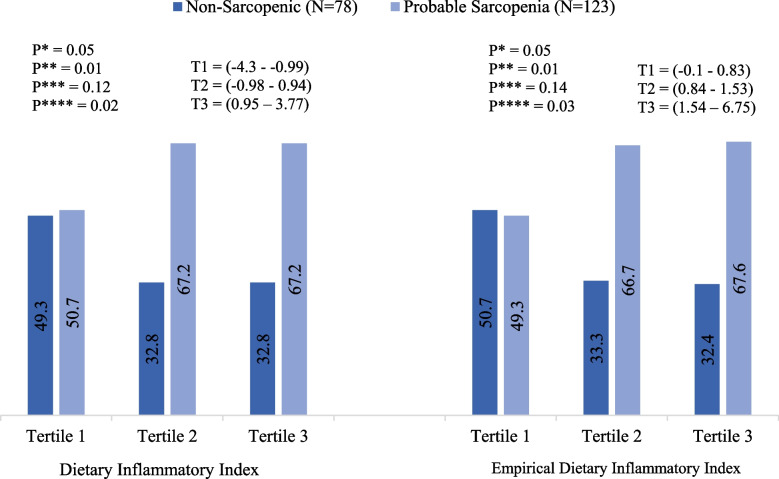
Association between PS and tertiles of DII and EDII. The bars indicate the percentages DII: Dietary Inflammatory Index. EDII: Empirical Dietary Inflammatory Index. T: Tertile. P*: Differences between tertiles. P**: Differences between T1 and other tertiles. P***: Differences between T3 and other tertiles. P****: Differences between T1 and T3. *P* ≤ 0.05; X^2^ test.

While mean DII scores varied significantly between non-sarcopenic (-0.3 (± 1.91)) and PS subjects (0.15 (± 1.85)) (*p* = 0.05), the association between EDII and PS remained insignificant (*p* = 0.85). Table 2 presents associations between components of the indexes across healthy and PS subjects. The probable-sarcopenic subjects had positive DII scores for saturated fats and thiamin, and a negative score for iron compared to healthy ones. None of the EDII components showed a significant association with PS. The prevalence of PS subjects was significantly reduced from 67.2% and 67.6% in the highest tertile of DII and EDII to 49.3% and 50.7% in the lowest tertile, respectively (Fig. 1).

**Table 2:** DII, EDII, and the components scores across probable and non-sarcopenic subjects

Variables (mean ± SD)	HGS				*P*-value*	*P*-value**
	Non-sarcopenic (*N* = 78)		Probable Sarcopenia (*N* = 123)			
	Mean	SD	Mean	SD		
**Dietary Inflammatory Index**	-0.3	1.91	0.15	1.85	0.1	**0.05**
Total Energy	0.003	0.11	-0.0007	0.1	0.79	0.73
Total Protein	0.002	0.01	-0.001	0.01	0.1	0.07
Total Carbohydrate	0.0003	0.06	0.0006	0.06	0.97	0.67
Total Fat	-0.02	0.17	0.01	0.2	0.22	0.59
Total Cholesterol	0.006	0.07	-0.003	0.06	0.3	0.13
Total SFA	-0.04	0.2	0.03	0.2	**0.02**	0.06
Total Iron	0.004	0.02	-0.002	0.02	**0.02**	**0.02**
Total B12	-0.001	0.06	0.002	0.06	0.72	0.72
Total MUFA	0.0001	0.005	-0.0001	0.005	0.81	0.79
Total PUFA	-0.006	0.2	0.0008	0.2	0.82	0.39
Total Fiber	-0.04	0.39	0.02	0.38	0.25	0.39
Total Magnesium	-0.04	0.3	0.02	0.27	0.13	0.18
Total Zinc	-0.02	0.18	0.01	0.18	0.24	0.4
Total Folate	-0.01	0.11	0.01	0.11	0.2	0.17
Total Niacin	-0.02	0.15	0.01	0.14	0.07	0.07
Total Riboflavin	-0.004	0.04	0.002	0.04	0.35	0.24
Total Thiamin	-0.01	0.06	0.006	0.05	**0.04**	**0.05**
Total Vitamin A	-0.01	0.24	0.005	0.23	0.59	0.44
Total Vitamin C	-0.009	0.24	0.002	0.25	0.77	0.59
Total Vitamin E	-0.01	0.25	0.004	0.24	0.65	0.27
Total Vitamin D	-0.03	0.26	0.01	0.26	0.28	0.28
Total Pyridoxine	-0.009	0.21	0.003	0.21	0.7	0.62
Total Selenium	-0.01	0.11	0.008	0.11	0.16	0.4
Garlic	0.01	0.23	-0.01	0.24	0.48	0.71
Onion	0.001	0.16	-0.003	0.18	0.87	0.86
Turmeric	-0.02	0.46	0.005	0.43	0.73	0.18
Pepper	-0.003	0.08	0.0008	0.07	0.73	0.18
Tea	0.009	0.32	-0.01	0.3	0.67	0.83
**Empirical Dietary Inflammatory Index**	1.36	1.17	1.42	0.91	0.69	0.85
Processed Meat	0.01	0.03	0.1	0.02	0.87	0.75
Other Fish	0.01	0.008	0.007	0.01	0.37	0.22
Red Meat	0.03	0.02	0.03	0.03	0.19	0.67
Organ Meat	0.0003	0.0008	0.0001	0.0003	0.12	0.08
Grains	1.1	1.12	1.14	0.9	0.78	0.94
Other Vegetables	-0.04	0.35	-0.38	0.26	0.42	0.11
Tomatoes	0.02	0.01	0.01	0.01	0.38	0.17
High Energy Beverages	0.01	0.03	0.02	0.07	0.11	0.13
Leafy Green Vegetables	-0.11	0.2	-0.09	0.07	0.24	0.32
Dark Yellow Vegetables	-0.02	0.01	-0.02	0.03	0.69	0.86
Fruit Juice	-0.003	0.01	-0.004	0.01	0.54	0.75
Snacks	0.42	0.007	-0.009	0.04	0.29	0.28
Tea	-0.05	0.05	-0.04	0.04	0.75	0.62
Pizza	-0.004	0.04	-0.003	0.007	0.46	0.48
Coffee	-0.05	0.04	-0.03	0.12	0.27	0.32

*DII* Dietary Inflammatory Index, *EDII* Empirical Dietary Inflammatory Index, *HGS* Handgrip strength, *SD* standard deviation, *SFA* Saturated Fatty Acid, *MUFA* Mono-Unsaturated Fatty Acid, *PUFA* Poly- Unsaturated Fatty Acid

^*^P ≤ 0.05, Student’s t-test; **P ≤ 0.05, Analysis of covariance (ANCOVA), adjusted for age, family number, gender, CVD medication, BMI, and physical activity

### Dietary inflammatory potential, hand grip strength, and confounders

Regarding the findings of multiple linear regression analyses (Table 3), there was a negative significant association between the DII score and HGS in the unadjusted model (adjusted R^2^ = 0.03, B = -0.37, *p* = 0.009), plus EDII score and HGS after adjusting for confounders (adjusted R^2^ = 0.52, B = -0.49, *p* = 0.04). Considering the adherence to indexes, those in the top tertile of DII (indicating a more pro-inflammatory diet) had a higher likelihood of PS in comparison with normal ones in both unadjusted (OR = 2.11, 95% (CI) = 1.05–4.24) and adjusted models (OR = 2.7, 95% (CI) = 1.25–5.8; OR = 2.49, 95% (CI) = 1.11–5.58). Besides, subjects in the second tertile of EDII were 2.29 times (95% (CI) = 1.03–5.07) more likely to have PS than those in the lower tertile in the adjusted model (Table 4).

**Table 3:** Multiple linear regression for the association of handgrip strength with DII and EDII

Variables		Adjusted R^2^	Unstandardized Coefficients	95% (CI)	*P*-value*
			B (SE)		
Dietary Inflammatory Index	Crude model	0.03	-0.37 (0.14)	(-0.65—-0.09)	0.009
	Model I^a^	0.46	-0.16 (0.11)	(-0.37 – 0.05)	0.14
	Model II^b^	0.51	-0.29 (0.24)	(-0.77 – 0.18)	0.22
Empirical Dietary Inflammatory Index	Crude model	-0.005	-0.07 (0.26)	(-0.59 – 0.44)	0.78
	Model I^a^	0.46	-0.23 (0.19)	(-0.61 – 0.15)	0.23
	Model II^b^	0.52	-0.49 (0.24)	(-0.95—-0.02)	0.04

^*^P ≤ 0.05

^a^ Adjusted for age and gender

^b^ Adjusted for age, family number, gender, CVD medication, Body Mass Index and physical activity

**Table 4:** Logistic regression: probable sarcopenia

Variables	Crude model	Model I ^a^	Model II ^b^
	OR 95% (CI)	OR 95% (CI)	OR 95% (CI)
Dietary Inflammatory Index model			
Tertile 1	1.00	1.00	1.00
Tertile 2	2.11 (1.05–4.24)^†^	2.31 (1.09–4.9)^†^	2.3 (1.05–5.12)^†^
Tertile 3	2.11 (1.05–4.24)^†^	2.7 (1.25–5.8)^†^	2.49 (1.11–5.58)^†^
P-trend	0.04	0.01	0.02
Empirical Dietary Inflammatory Index model			
Tertile 1	1.00	1.00	1.00
Tertile 2	2.06 (1.02–4.15)^†^	2.67 (1.24–5.71)^†^	2.29 (1.03–5.07)^†^
Tertile 3	2.15 (1.07–4.33)^†^	2.35 (1.1–5.00)^†^	1.91 (0.85–4.25)
P-trend	0.03	0.03	0.10

^†^ P ≤ 0.05

^a^Adjusted for age and gender

^b^Adjusted for age, family number, gender, CVD medication, Body Mass Index and physical activity

## Discussion

For all we know, this is the first study attempt to investigate the association between the inflammatory potential of the diet and PS among older adults by both DII and EDII regardless of comparing the results of both indexes with each other. The findings of this study represented that a more pro-inflammatory diet doubled the odds of PS in older adults even after adjusting the association for confounders. The cutoff values of EWGSOP2 were used in the current study which has been reported to be good indicators in Iranian populations [31].

The number of studies on the association between dietary inflammatory potential and muscle weakness is limited and findings are a point of contention. Similar to our study, an increased odds of low grip hand was found by Kim et al. in older individuals adhering to a pro-inflammatory diet [32]. As reported by Laclaustra et al., there was a link between a pro-inflammatory diet and frailty in older adults [17]. The possibility of osteosarcopenic obesity increased in postmenopausal Korean women with high DII scores through the findings of Park et al [21]. Unlike the association between dietary inflammatory potential, evaluated by DII, with greater risk of sarcopenia, Bagheri et al. failed to show a significant difference between abnormal HGS and tertiles of DII [22]. Besides, the suggested linkage between energy-adjusted DII and abnormal HGS by Cervo et al. was in significant as well [23]. These conflicts might be explained by the dissimilarity of dynamometers and populations among studies. It appears that additional data is required to give insight into the association between dietary inflammatory indexes and muscle strength.

In the present study, PS subjects consumed more saturated fats and had a less dietary intake of anti-inflammatory nutrients compared to healthy people. Based on the findings, it seems that people with a high possibility of sarcopenia consumed less fruit and vegetable as the main sources of these anti-inflammatory nutrients concerning subjects with normal HGS. Consistent with our study, Hashemi et al. showed that older adults with high adherence to the Mediterranean diet had low odds of sarcopenia [33]. Participants with a high probability of sarcopenia consumed less fruit and vegetable with less adherence to Healthy Eating Index, Dietary Quality Index, and Mediterranean Diet in several studies [25, 34–36]. Although the levels of inflammatory markers have not been assessed in the current study, it has been remarked that higher hs-CRP is directly associated with oxidative stress which has been introduced as a major underlying mechanism of sarcopenia pathogenesis in previous studies [37, 38]. On the one hand, rising pro-inflammatory cytokine levels like TNF-α, IL-6, and hs-CRP happen through aging which exacerbates the inflammatory process, and consequently, accelerates muscle weakness [39]. Moreover, saturated fats provoke inflammatory responses through the NF-kB pathway. Thus, contrary to mono- or polyunsaturated fatty acids (MUFAs or PUFAs) as anti-inflammatory nutrients, high consumption of saturated fats might play role in impaired muscle strength [40, 41]. On the other hand, inflammatory mediators downregulate insulin and insulin-like growth factor-1 (IGF-1) which decrease muscle protein synthesis [42]. In this case, muscle atrophy tends to occur. A decrease in muscle mass might impair muscle strength as well, unnecessarily in a linear relationship. Notably, muscle weakness could occur rapidly compare to muscle mass decline [5, 43–46]. Nevertheless, the findings of the different studies aroused much controversy on the association between muscle mass and muscle strength and more investigations are required to clarify this association.

Though these findings were novel in this concept, PS was distinguished by using the recent definition of EWGSOP, and subjects were randomly selected from Tehran’s all regions which provides a good portrayal of Tehran’s older adults, this study has some limitations. Primarily, the squeeze dynamometer used here has a lower accuracy relative to digital ones. This is a cross-sectional study in that the serum concentration of inflammatory markers wasn’t measured and unable to verify any causality as well as it cannot specify the role of diet in PS precisely. Since the FFQ was used for dietary intake assessment, we can’t ignore the recall bias and over-report or under-report of participants. Finally, some of the DII components were not included in the calculation of total DII in this study which may cause underestimation of the relationship, although, Shivappa et al. reported that including at least 28 dietary parameters for its calculation did not drop DII’s predictive ability [12].

## Conclusion

In conclusion, adherence to a diet with greater inflammatory potential might significantly impact the possibility of sarcopenia in older adults. These results are in line with recent recommendations to substitute healthy foods and emphasize the consideration of dietary choices in elderly health status. These findings warrant confirmation in further well-designed studies.

## Data Availability

Data described in the manuscript, code book, and analytic code will be made available by the corresponding author upon request pending.

## Abbreviations

HGS: Handgrip strength
PS: Probable sarcopenia
DII: Dietary Inflammatory Index
EDII: Empirical Dietary Inflammatory Index
EWGSOP: European Working Group on Sarcopenia in Older People
BMI: Body mass index
WHR: Waist to hip ratio
WHtR: Waist to height ratio
MUAC: Mid-upper arm circumference
FFQ: Food frequency questionnaire
MUFAs: Monounsaturated fatty acids
ANCOVA: Analysis of covariance
PUFAs: Polyunsaturated fatty acids
IGF-1: Insulin-like growth factor-1
